# Design Optimization and Structural Performance Evaluation of Plate Girder Bridge Constructed Using a Turn-Over Process

**DOI:** 10.3390/ma10030283

**Published:** 2017-03-13

**Authors:** Gi-Ha Eom, Sung Jae Kim, Tae-Hee Lee, Jang-Ho Jay Kim

**Affiliations:** School of Civil and Environmental Engineering, Yonsei University, Seoul 03722, Korea; gheom@hanmail.net (G.-H.E.); spc4000@naver.com (S.J.K.); saintlth@yonsei.ac.kr (T.-H.L.)

**Keywords:** two- or three- girder plate bridge, precasted girder, turn over construction, preflex method

## Abstract

A recent trend in bridge construction has been the optimization of the cost-to-performance ratio. The most effective way to optimize the cost-to-performance ratio is to maximize the efficiency of the superstructure. Currently, many bridge engineers and designers favor two- or three- girder plate superstructures, due to their cost advantages. However, research on the performance enhancements of the I-type girder in two- or three- girder plate bridges is lacking. One of the most important performance improvement technologies for the I-type girder is the “preflex” method. In the preflex method, the specimen is inverted during the construction process to apply prestressed cambering to the specimen by using self-weight. However, a problem with the preflex construction method is difficulty with inverting the girder/plate system during the concrete curing process. Therefore, a new inverting system called Turn-Over (TO) wheel was proposed. Using TO wheels, wider variations to the I-type girder design can be achieved. Using this TO construction method, various cross sectional designs of girder plate systems can be considered due to its easiness in inverting the girder/plate system. In this study, the location of concrete confinement sections between the steel I-beams and concrete plates was varied in an I-girder cross-sectional design. Design parameters included effective height, flange thickness, flange width, confining concrete section width, etc. From this study, the optimum cross-sectional design of the I-girder/concrete plate system was achieved. Then, a single 20 m TO girder/plate system and two 20 m TO girder bridges were constructed and tested to evaluate their performance. From the test, failure behavior, load carrying capacity, crack pattern, etc., are obtained. The results are discussed in detail in this paper.

## 1. Introduction

As urbanization accelerates and city populations rapidly increase, transportation of people and merchandise must become more efficient. Bridge construction plays a vital role in the urban transportation system, in order to improve transport systems and infrastructure. Recently, as bridge construction technologies advance, construction efficiency, sustainable maintenance, and construction cost optimization are becoming important issues in bridge construction. In order to resolve these issues, many studies have been conducted to optimize the cost-to-performance ratio. Among those concerning bridge technologies, the superstructure of a bridge was identified as a critical aspect in maximizing bridge efficiency [1,2,3].

**Figure 1:**
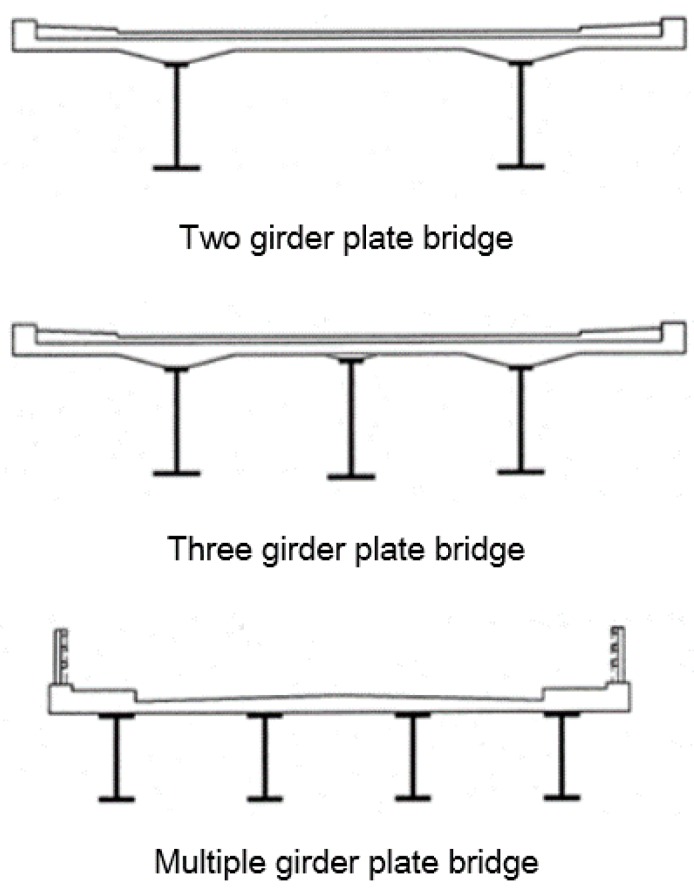
Number of I-girders in plate girder bridges.

Generally, the bridge superstructure can be divided into two categories. One type is a closed system and the other is an open system, such as a box-type girder or plate-type girder system, respectively. The box-type girder system is advantageous in torsion and durability, but it is disadvantageous in terms of construction costs. The plate-type girder system is disadvantageous in terms of durability and constructability, but it is advantageous in relative construction and maintenance costs compared to the other girder systems. Currently, since the most important issue in bridge construction is cost-to-performance efficiency, many bridge engineers and designers favor a plate-type girder system, due to the cost advantages [4,5,6]. A plate girder bridge is a bridge constructed by placing a concrete plate on steel or concrete I-type girders. Therefore, a plate girder bridge is usually a precast-type that can save construction time, and works by transporting and placing the precasted girders on top of the pre-constructed bridge piers on-site. There are two types of plate girder bridges. One type is the multiple girder plate bridge, and the other is the two- or three- girder plate bridge as shown in Figure 1.

Previously in Korea, multiple girder plate bridges were constructed more frequently, because Korean bridge engineers and designers did not have sufficient knowledge and experience in designing two- or three- girder plate bridges. Also, compared to multiple girder plate bridges, two- or three- girder plate bridges tended to be less safe [7,8]. However, due to recent advancements in bridge technologies, construction materials, and precast construction methods, two- or three- girder bridges are becoming more popular. More specifically, two- or three- girder plate bridges have a much simpler structural behavior, better cost-to-performance ratio, and easier structure maintenance, due to a reduction in the number of girders [8].

**Figure 2:**
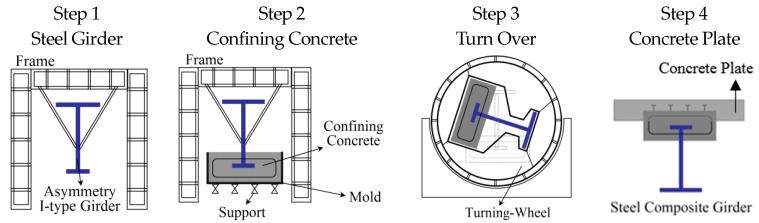
Construction procedure of steel composite girder applied by Turn-Over process (TO).

**Figure 3:**
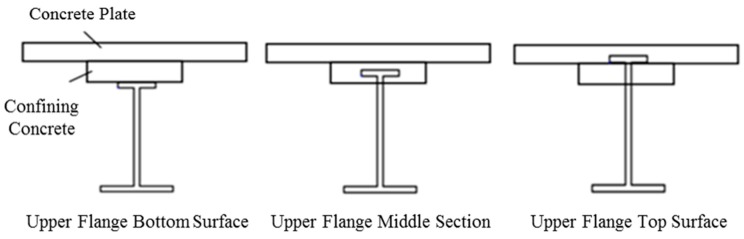
Location of the confining concrete section in the cross section.

In technologically developed countries, such as France, Germany, Switzerland, Japan, and Korea, bridge engineers and researchers attempted to maximize the constructability, safety, serviceability, and durability of two- or three- girder plate bridges. Jeon et al. conducted research on life cycle cost (LCC) optimization in the design of main girders to improve constructability and durability of two- or three- girder plate bridges [9]. Also, Yun et al., Lin et al., and Park et al. tried to improve safety and durability of two- or three- girder plate bridges by focusing on its redundancy [10,11,12,13]. In addition, high performance and high strength steel member developments for two- or three- girder plate bridges have been performed by Coelho et al., Ricles et al., and Yong et al. [14,15,16]. Even though many different types of research have been conducted on two- or three- girder plate bridges, the literature review shows that studies on enhancements of the I-type girder improvement is lacking. However, a breakthrough in performance for the I-type girder was proposed by Lipski [17], called the “preflex” method. In the preflex method, pre-deflection is applied to the steel I-type girder using the dead load of the concrete plates by inverting the girder/plate system during the precast construction process as shown in Figure 2. The preflex method is equivalent to the prestressing method in concrete girders where the initial cambering deflection is applied to the girder to increase the load carrying capacity and plastic deflection serviceability. Using the preflex method during construction, the I-type girders and two- or three- girder bridge systems became much safer and more stable, almost equivalent to multiple girder systems. However, variations to the I-type girders constructed using the preflex method were limited due to the difficulty in inverting the girder/plate system during the curing process. Therefore, in this study, a new inverting system called Turn-Over (TO) wheel is proposed to make the preflex girder construction simpler and quicker. In the TO system, two ends of the girder/plate system are initially placed into the TO wheels. Then, when the concrete plate hardens sufficiently, the wheels are turned to invert the system as shown in Figure 2 (step 3). It is important to note that the TO method is equivalent to the preflex method, except that the inverting of the girder/plate specimen is easier in the TO method through the use of the TO wheels. Using the TO system, more variations to the I-type girder design can be achieved due to the simplicity of the precast construction. In this study the structural performance and failure behavior of various cross-sectional designs of I-type girders constructed using the TO system are evaluated. Specifically, the confining concrete section between the upper flange of the I-type steel. The concrete plate is placed at the top, middle, and bottom section of the upper flange of the I-type steel to determine the best location of the girder/plate interface, as shown in Figure 3. Then, a single 20 m TO girder and two 20 m TO girder bridge are tested. The details of the TO system girder designs, test specimens, and test results are discussed in this paper.

## 2. Turn-Over Construction Method

### 2.1. Basic Theory

The Turn-Over (TO) method is used to reduce steel section size in a steel I-beam by applying initial deflection using distributed self-weight of the confining concrete section and concrete plate cast on the top flange section. The application of the concrete self-weight cambering achieves reduction of stress in the member during its service life without any additional treatment, such as thermal prestressing, tendon prestressing, etc. [18,19,20,21,22,23,24,25].

The TO method is comprised of four main steps, shown in Figure 2. Step 1 is the overhanging of an asymmetric I-section steel member on a frame to start the manufacturing process. In this step, the initial bottom section, which will ultimately be turned over as a top section of the girder. Step 2 is casting of the confining concrete section at the bottom flange of the overhanging I-section member. Once the confining concrete sufficiently hardens, the distributed self-weight applies a deflection on the I-section steel member. In step 3, the whole member is turned upside down, where the bottom section goes to the top and the top section comes down to the bottom. The process is facilitated by using the turning wheel instead of manually turning over the member, which reduces the construction time and effort. Once the member has been turned over, in step 4 a concrete plate is cast on top of the confining concrete section to complete the process. The cambering deflection applied to the steel I-beam from the self-weights of the confining concrete section is returned back to “zero” deflection when the dead load of the bridge is applied. In step 4, the best composite action between the concrete plate and the confining concrete section is selected by determining the optimal location of the confining concrete section with respect to the top flange of the I-section.

**Figure 4:**
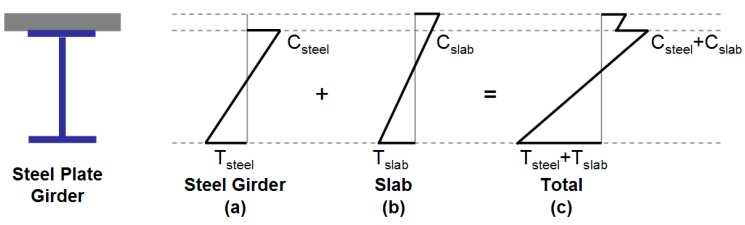

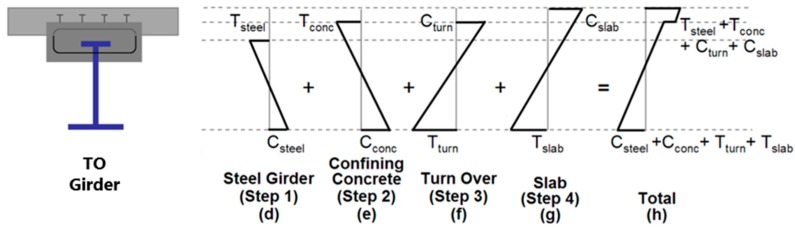
Strain profiles along the cross section height of the ordinary plate girder and TO girder.

The comparison of strain profiles along the cross section of the ordinary plate girder and the TO girder is shown in Figure 4. In the ordinary steel girder, the addition of stresses from the self-weight of the girder (Figure 4a) and the concrete plate (Figure 4b) would result in summed tensile and compressive stresses at the bottom and top of the cross section, respectively, as shown in Figure 4c. Figure 4d–f shows the stress profiles occurring in the TO girder before casting of a plate. As shown in Figure 4a,d, stresses in the cross section between the ordinary and the TO steel girders are similar in magnitude, but compression and tensile stresses are in opposite directions. Therefore, in the TO girder, the bottom and top sections are under compressive and tensile stresses, respectively, due to the upside-down characteristic of the girder at the initial manufacturing process. When the confining concrete section is placed at the top flange of the TO girder, additional tensile and compressive stresses are applied to top and bottom of the cross section, respectively. As the TO girder is turned over using the turning wheel, top and bottom section stresses are also turned over and change direction, as shown in Figure 4f. When the plate is cast above the confined concrete section and the top flange section of the TO girder, additional compressive and tensile stresses are applied to the top and bottom flanges, respectively, as shown in Figure 4g. When all of these stresses are added together, the TO girder has the total tensile and compressive stresses at the bottom and top sections, respectively shown in Figure 4h. It is important to note that the magnitude of the tensile and compressive stress is much less than that of the ordinary steel girder, which is the reason for the steel reduction in the TO girder. Also, because of the upward shifting of the neutral axis due to the confining concrete and flange sections of the girder, the optimal cross section or the minimal steel design can be achieved for the TO girder. Design equations used in Figure 4 are shown in Table 1.

The comparison of the amount of steel required for an ordinary steel girder and the TO girder is tabulated in Table 2. As shown in Table 2, the steel required for the confined section located at the bottom flange of the TO girder with a confining concrete section width and height of 400 mm and 1000 mm, respectively, shows no significant difference compared to that of an ordinary steel girder. However, with respect to the top flange, the required steel for the TO girder is reduced by 85.94% compared to that of an ordinary steel girder. The reduction in steel in the TO girder results in savings of material costs and construction time from the precasted construction using the TO method.

### 2.2. The Optimum Cross Section Determination

In order to obtain the optimal cross section design for the TO girder, the following construction parameters have been considered: (1) Confining the concrete section location; (2) Cross-section weight and steel ratios; (3) Span-to-depth ratio.

#### 2.2.1. Confining the Concrete Section Location

In order to determine the optimal composite action between the confining concrete and plate sections, the location of the confining concrete section in the cross section is varied, as shown in Figure 3. The effective height of the bridge cross section is maintained at 3200 mm for all three cases shown in Figure 3. The cross section dimensions of the confining concrete section are 1000 mm in height and 400 mm in width. The three locations considered for the confining concrete section are as follows.

1. The confining concrete section is located between the bottom surface of the plate and the top flange section surface of the I-steel member.
2. The top flange of I-steel member is embedded in the confining concrete section and the confining concrete section is attached to the bottom surface of the plate.
3. The confining section is attached to the bottom surface of the top flange section of the I-steel member with the top flange section embedded in the plate.

With the confining concrete section location parameter as the top surface, center, and bottom surface of the top flange section of the I-steel member, this study focuses on the stress and steel ratios of a TO girder bridge. When the confining concrete section is located at the top surface of the top flange of I-steel member, the compressive forces acting on the confining concrete section is large enough to require a larger width of the confining section. The calculated amount of steel needed for this setup is approximately 50 t. When the top flange section is embedded in the confining concrete section, inverting of the system becomes easier and the required amount of steel is 50.2 t. Finally, when the confining concrete section is placed at the bottom surface of the top flange section of the steel I-beam, the compressive force acting on the confining concrete section is increased due to the effective height reduction of the cross section and the required steel amount is approximately 51.0 t. Also, in order to implement this setup, dowel studs and complex formwork are required, making the construction much more difficult. Also, since the effective height of the girder bridge is increased, the required steel amount is increased. However, since the difference in the required steel amount for all three cases is nearly equal, the case with the top flange section embedded in the confining concrete section is selected for its construction simplicity, better structural stability, smoother force transfer, better embedment connection of the steel I-beam, etc.

#### 2.2.2. Required Steel and Stress Ratios for Various Cross-Sectional Parameters

Once the confining concrete section location is selected, the required amount of steel and concrete section area are calculated based on 9 different cross-sectional parameters as follows: the effective height (*H*), the top and bottom flange thicknesses and widths (*T*ft, *T*fb, *W*st, and *W*sb), the confining concrete section thickness and width (*T*c and *W*c), and the distance between confining concrete and top flange upper surfaces (*H*c) as shown in Table 3. In Table 3, the upper and lower values for all of the parameters are tabulated, obtained from design equation calculations of required strength. Based on these upper and lower values, the design of the TO girder cross-section is performed.

**Figure 5:**
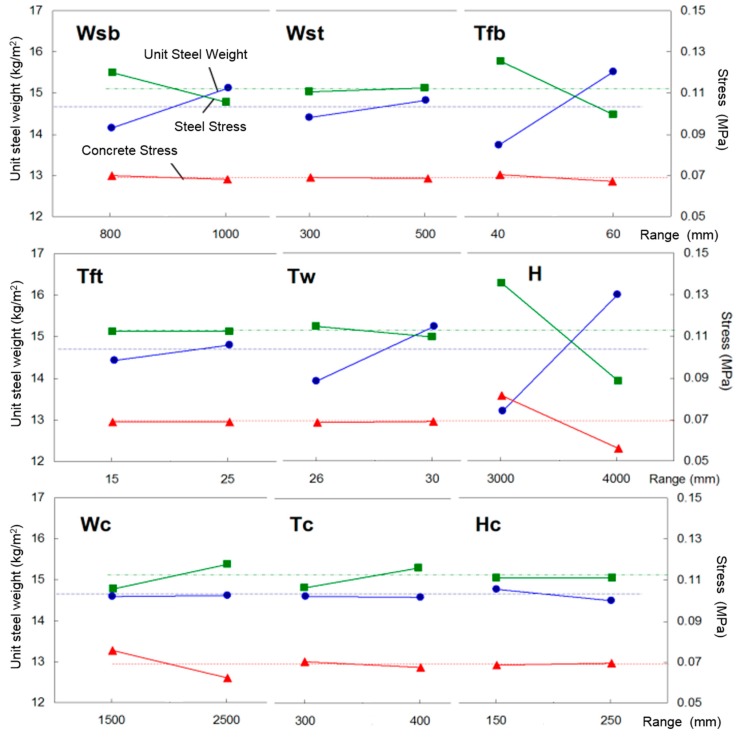
Steel weight, steel and concrete stress by cross section area.

The total number of possible parametric variables cases are 2^9^ = 512. However, if the Determination of Experiment (DOE) method is used, then the parametric variable cases can be reduced to one eighth of 512, i.e., 64 cases. Also, in Korea for a 50 m span girder, a general steel weight per unit area is less than 200 kg/m^2^ and the allowable stress limit is 90% of the actual stress. Therefore, these values are used as the criteria in the analysis. From the analysis results, the required steel weight, steel stress and concrete stress were plotted, as shown in Figure 5. Depending on the slope of the curve between the upper and lower values of the parameter, the parameter’s sensitivity to the cross-sectional design can be observed. In Figure 5, the dotted lines indicate the average values of required steel weight, steel stress, and concrete stress. From the parametric study, the order of parameter importance in required steel amount is as follows: the effective height (*H*) > the bottom flange thickness (*T*fb) > the web thickness (*T*w). With respect to the steel stress, the order is as follows: the effective height (*H*) > the bottom flange thickness (*T*fb) > the bottom flange width (*W*sb). Finally, with respect to the parameter effect on stress applied to concrete section, the importance of order is as follows: the effective height (*H*) > the confining concrete section width (*W*c).

In conclusion, the effective height most significantly affects the required steel weight as well as the steel and concrete stresses on the cross section. Since the number cases from nine different parameters turns out to be 64 cases, the different parameter combinations give slightly different result trends. Therefore, in the case of *H*c parameter, the required unit steel weight at the upper value is less than the lower value, due to the nonlinear trend in the analysis result.

## 3. Performance Evaluations by TO Girder Static Testing

**Figure 6:**
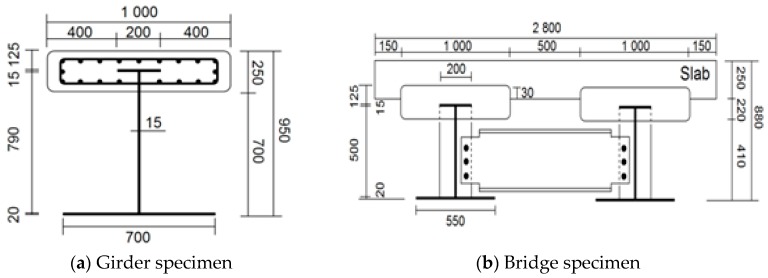
Cross section dimensions. (**a**) Girder specimen; (**b**) Bridge specimen. (Unit: mm).

In order to evaluate the performance of the girder built by the TO method, a 20 m TO girder specimen and a two-main TO girder plate bridge specimen with a plate section are tested. The cross section dimensions of these two specimens are shown in Figure 6a,b.

### 3.1. Expeimental Details

**Figure 7:**
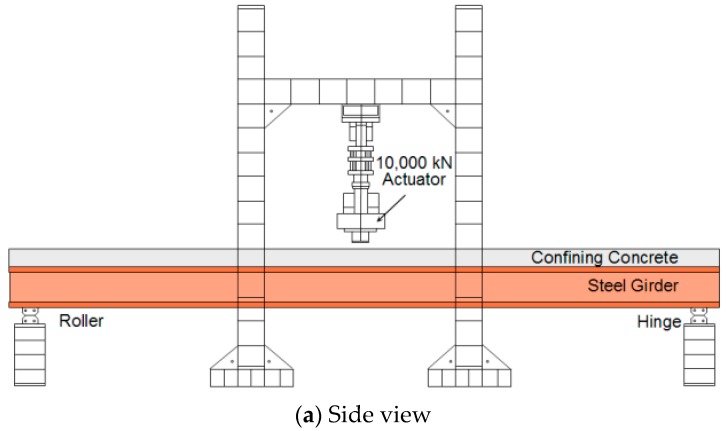

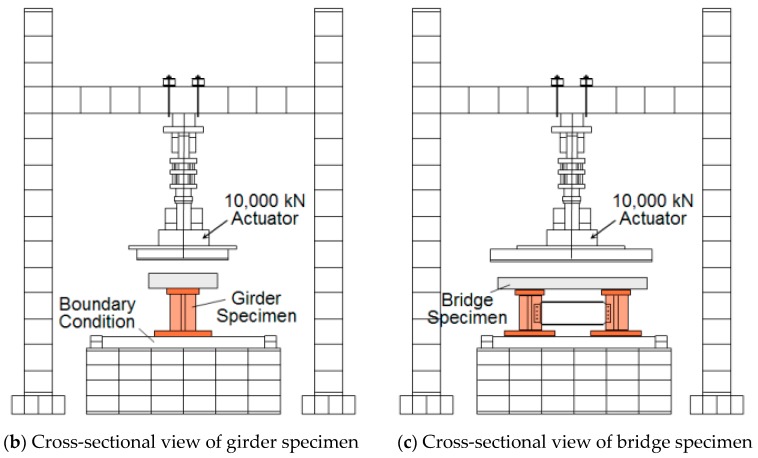
Schematic drawings of the bending test setup, (**a**) Side view; (**b**) Cross-sectional view of girder specimen and (**c**) Cross-sectional view of bridge specimen.

The TO girder and bridge specimens with a central location for a confining concrete section were statically tested to evaluate their load carrying capacity. As shown in Figure 6, the width of the girder and the plate were 1.0 and 1.5 m, respectively. HSB-600 reinforcements and 50 MPa 28 day compressive strength concrete were used to design the specimens. However, due to the testing site and casting condition limitations, the 100 × 200 mm cylindrical concrete specimen’s 28 day compressive strength data at 30 MPa and 27 MPa were only available to cast the confining concrete and the plate sections, respectively. As shown in Figure 6b, the top flange of the I-steel member is embedded in the confining concrete section and approximately 30 mm of the confining concrete section is embedded in the plate section. Also, the two-main girder/plate were connected by a stiffener connection using bolt attachments. The specimens were tested in a simply supported setup with three-point loading. For the application of the load, UTM with 10,000 kN capacity was used. In order to firmly set the specimen on the supports, the load was applied up to 300 kN with a 100 kN force loading increment before a displacement controlled loading was applied at a rate of 0.1 mm/s. The schematic drawings of the specimen setup are shown in Figure 7. A center span deflection was measured using a 250 mm LVDT. The top and bottom flange surface strains and mid-height web strains were measured using strain gauges. Embedded strain gauges were also placed in the confining concrete section and on reinforcement surfaces of the plate section.

### 3.2. Results and Discussion

#### 3.2.1. Load-Deflection Relationship

**Figure 8:**
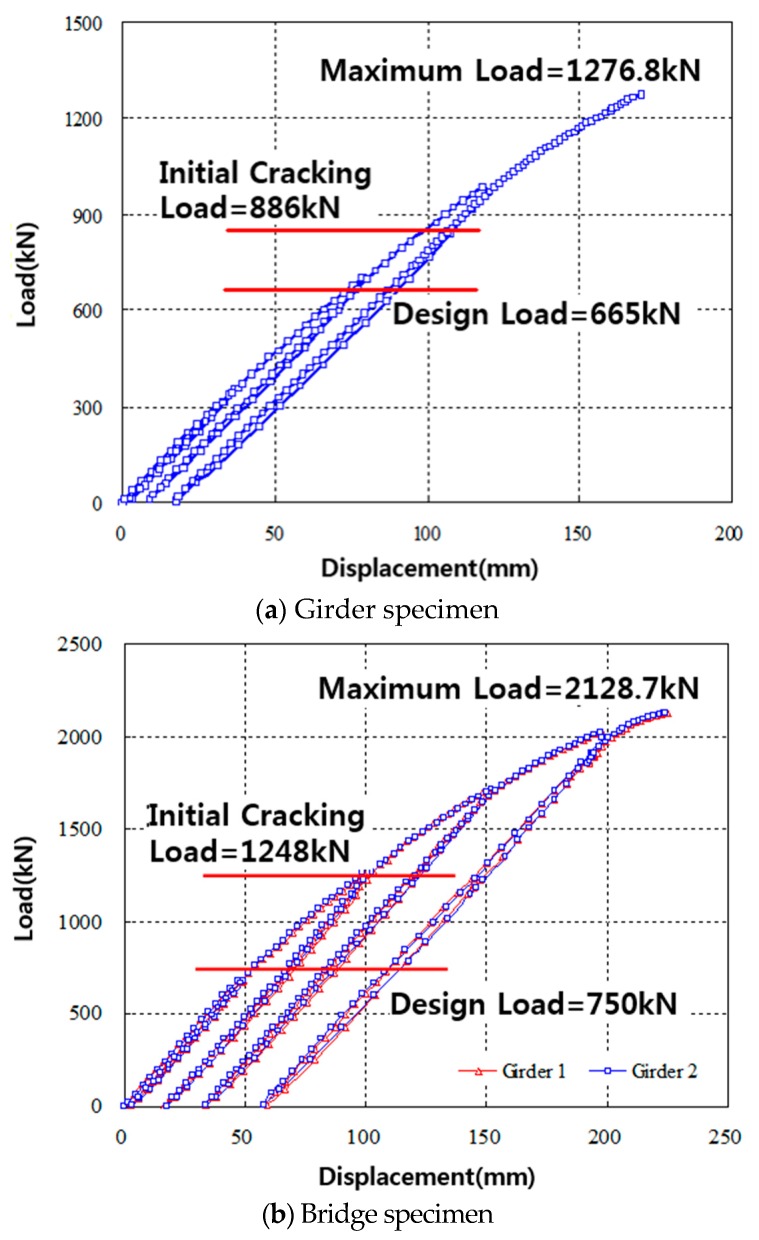
Load-deflection relationships measured at the center of the span, (**a**) Girder specimen and (**b**) Bridge specimen.

The load-deflection relationships measured at the center of the span for the girder and bridge specimens are shown in Figure 8a,b respectively. The girder and bridge specimens initially cracked at loads of 886 kN and 1248 kN, respectively, with the corresponding center span deflection of 111.1 and 145.5 mm, respectively. These initial cracking loads were approximately 1.3 to 1.6 times greater than the design cracking load of 665 kN and 750 kN for the girder and bridge specimens, respectively. After initial cracking of the specimens, stiffness reduction occurred in both specimens at maximum loads of 1276.8 kN and 2128.7 kN with the corresponding center deflections of 170.4 and 220.4 mm for the girder and bridge specimens as shown in Figure 8a,b respectively. In Figure 8b the load cycles show were conducted as follows. The load was applied until initial cracks formed, then the specimen was unloaded. Then, the specimen was reloaded until the load reached approximately twice the design load, at which point, the specimen was unloaded. Finally, the specimen was reloaded until significant macro-damage occurred, which can be considered as an ultimate load. This load cycle was used to clearly understand the specimen failure behavior at initial cracking stage, service stage, and ultimate failure stage.

**Figure 9:**
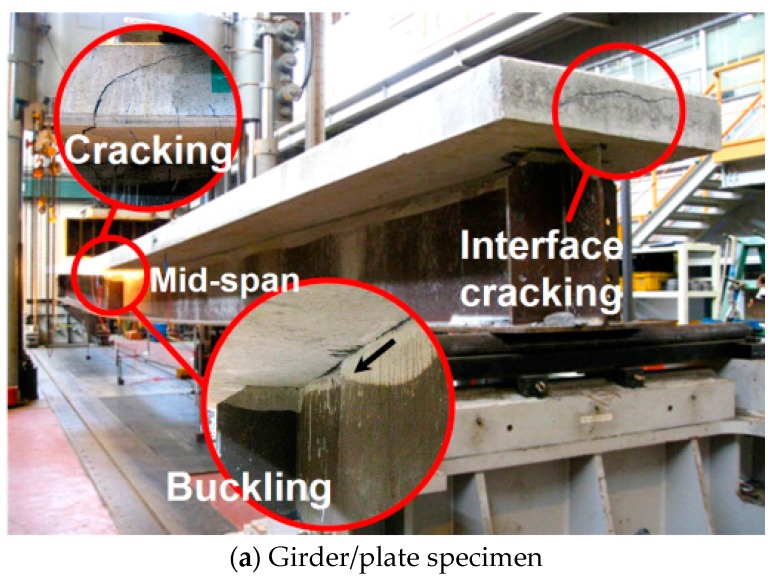

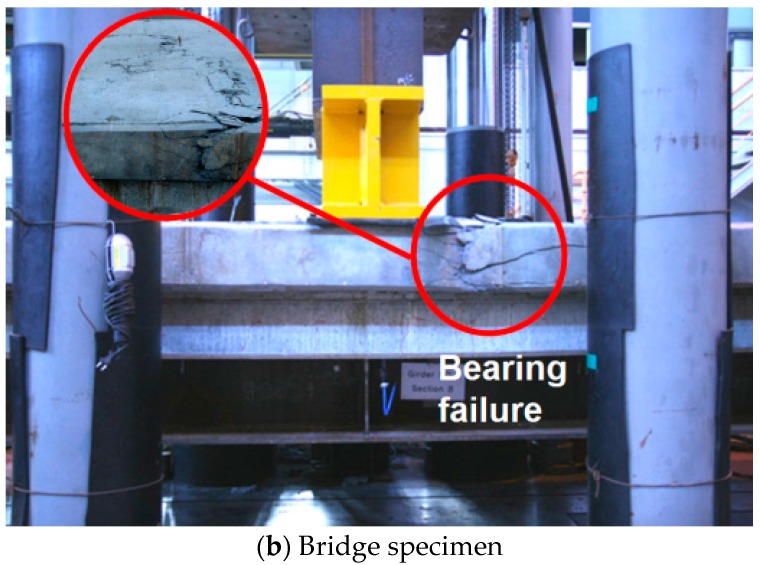
Failure pattern of girder and bridge test specimens, (**a**) Girder/plate specimen and (**b**) Bridge specimen.

Since the load-deflection data obtained from both the girder and bridge specimens are similar, it is safe to conclude that the stress distributions in the bridge specimens are even and symmetrical. In both girder and bridge specimens, both buckling and interfacial cracking failures occurred. However, only in a bridge specimen was there bearing failure (e.g., concrete spalling above the support), as shown in Figure 9. Because of the buckling and interfacial cracking failures, the ultimate loads could not be measured. However, since the maximum applied load was approximately 1.9 to 2.8 times greater than the design load, it is safe to conclude that the load carrying capacity of the TO girder and TO girder bridge is sufficient for practical applications.

#### 3.2.2. Load-Strain Relationship

**Figure 10:**
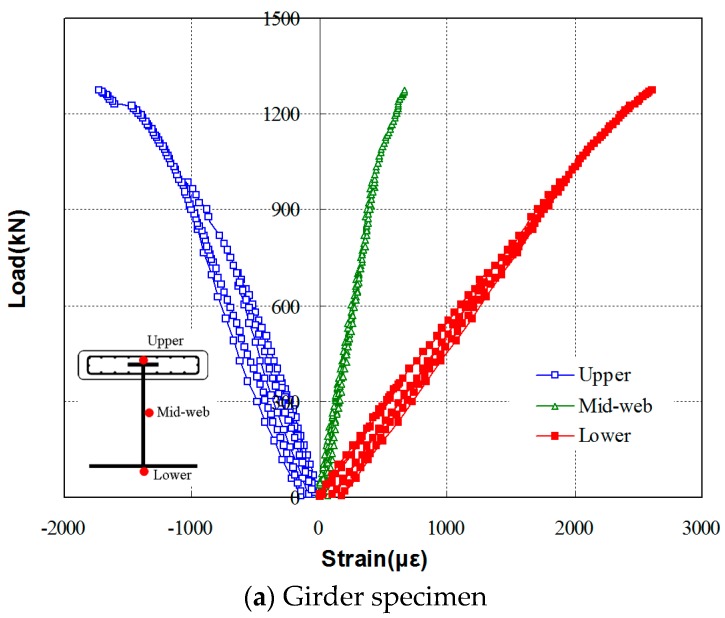

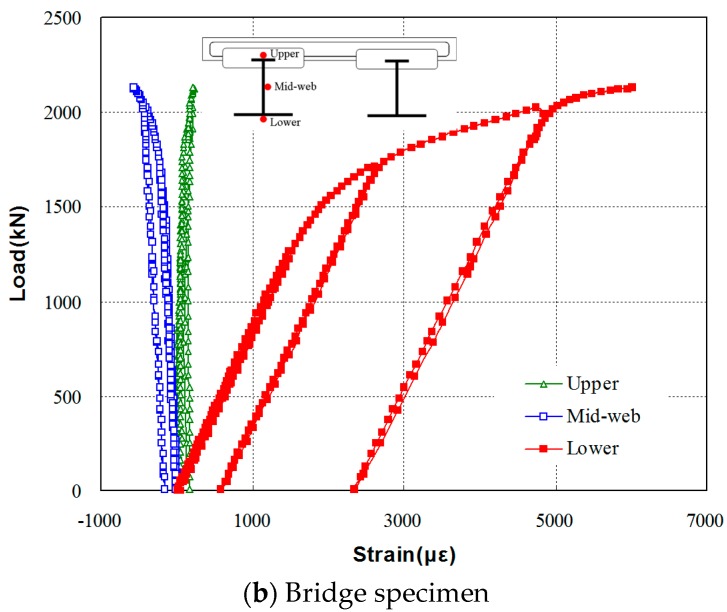
Load-strain relations at the center span of the girder and bridge specimens, (**a**) Girder specimen and (**b**) Bridge specimen.

The load-strain relations at the center span of the girder and bridge specimens are shown in Figure 10a,b respectively. For the girder specimen, the initial cracking at the confining concrete section occurred approximately at a load of 900 kN, after which the non-linear load-strain relationship was observed. At this cracking load, the confining concrete section cracked, and the cracks propagated as the strain of the steel girder also correspondingly increased. Figure 9a shows a photo of local buckling failure, which occurred slightly after the confining concrete section cracked, indicating that the interfacial cracking caused this local buckling. Additionally, the interface failure of confining concrete and the I-steel girder is likely due to the compressive strength of concrete used to manufacture the specimens, which was lower than the required design compressive strength. However, the load-strain relationships obtained from the tests showed that the tensile strain of the bottom flange section exceeded 2500 με and the top flange had approximately 50% of the strain measured at the bottom flange. The relatively small compressive strain in the steel member is likely due to the confining concrete section cast at the top flange section. Due to the increased compressive stress resistance capacity of the section above the neutral axis, a smaller compressive force needed to be resisted by the steel section above the neutral axis, thereby reducing the size of the top steel flange section needed for the girder.

In the bridge specimen, initial cracking occurred at a load of approximately 1300 kN, after which a non-linear behavior was observed. Due to the confining effect from the confining concrete section, a very low strain of 580 με was observed at the web and top flange sections of the I-steel member with high strain values.

**Figure 11:**
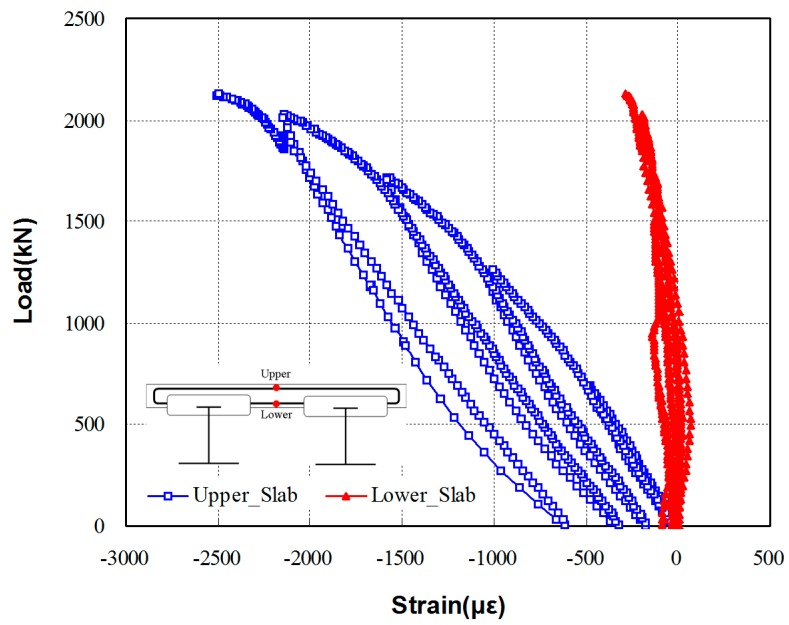
Load-strain relationship measured from the plate section of the bridge specimen.

Overall the specimen showed ductile behavior of the bottom flange section. As shown in Figure 11, both the top and bottom flange sections showed compressive stresses in the bridge specimen. The load-strain relationship measured from the plate section of the bridge specimen is shown in Figure 11. Both the top and bottom surfaces of the plate section showed compressive stresses, indicating that large compressive stresses occurred in the top section of the specimen. The test results indicate that the TO girder bridge specimen shows a good composite behavior between the plate and the girder, having a low effective height with strong compressive stress resisting capacity of the specimen. This indicates that the overall steel usage for the two- or three- main girder plate bridges can be significantly reduced by using the TO girders, proving its advantages for construction reductions in costs and time.

## 4. Conclusions

In this study, a modified precast construction method called Turn Over (TO) was proposed for construction efficiency improvement for turning over an I-section steel girder. A full-scale 20 m single girder specimen and a full-scale 20 m two-girder plate bridge specimen were used to investigate the optimum cross section design and the confining concrete section location, and to evaluate the structural performance. Based on the study results, the following conclusions were drawn.

- The ordinary I-section steel girder and proposed TO girder with a confining concrete section and a height of 3250 mm were compared. With respect to saving steel in the I-beam, the required steel area for the TO girder was reduced by over 13.8% than that of ordinary steel girders. Also, due to the simplicity of precast inverting construction using the TO method, the savings on manufacturing costs are significant. Therefore, there are significant economic benefits in using a TO girder over an ordinary steel girder.
- In order to investigate the optimal cross section for a TO girder, parametric studies of the confining concrete section location, cross-section weight, and stress ratios were carried out. The optimal location of the confining concrete section was selected as the flange of the I-beam. It was embedded in the section for its benefits in performance enhancements and construction simplicity. The sensitivity of the parameters with respect to steel weight and applied stresses are in the order of height, bottom flange thickness, and top flange thickness.
- A full-scale 20 m TO girder and bridge system were tested. From the static test results, initial crack of the TO girder and bridge system behaved elastically and occurred after reaching the design loads. Since the maximum applied load was approximately 1.9 to 2.8 times greater than the design load, the steel I-beam and concrete plate girder system constructed using the TO method can be assumed to have sufficient safety and load carrying capacity.
- From the TO manufacturing, the steel I-beam showed well distributed deflection from the application of self-weight of the confining concrete section. Since the failure load of the TO girder system increased by implementing the confining concrete section, the efficient composite behavior between the I-section steel girder, confining concrete, and concrete plate can be assumed.
- Analysis of the study results showed that the proposed TO method can be applied to practical designs as an improved precast construction method. However, additional experiments with other parametric variations of the TO girder need to be carried out. (i.e., buckling, interface bonding, etc.)

## Figures and Tables

**Table 1:** Stresses induced in the I-beam from Turn-Over (TO) method.

Step-Title	Manufacturing Step	Top Flange Stress	Bottom Flange Stress
Steel I-beam self-weight	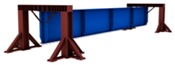	$f_{\mathrm{steeltop}}=\frac{M_{\mathrm{steel}}}{S_{\mathrm{steeltop}}}$	$f_{\mathrm{steelbottom}}=\frac{M_{\mathrm{steel}}}{S_{\mathrm{steelbottom}}}$
Confined concrete self-weight	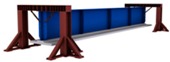	$f_{\mathrm{steeltop}}=\frac{M_{\mathrm{con}}}{S_{\left(s+c\right)\mathrm{top}}}$	$f_{\mathrm{steelbottom}}=\frac{M_{\mathrm{con}}}{S_{\left(s+c\right)\mathrm{bottom}}}$
Turn-Over process	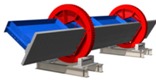	$f_{\mathrm{steeltop}}=\frac{2M_{\mathrm{steel}+\mathrm{con}}}{S_{\left(s+c\right)\mathrm{top}}}$	$f_{\mathrm{steelbttom}}=\frac{2M_{\mathrm{steel}+\mathrm{con}}}{S_{\left(s+c\right)\mathrm{bottom}}}$
Upright position	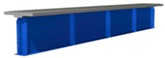	$f_{\mathrm{steeltop}}=\;\frac{M_{\mathrm{steel}}}{S_{\mathrm{steeltop}}}+\frac{M_{\mathrm{con}}}{S_{\left(s+c\right)\mathrm{top}}}-\frac{2M_{\mathrm{steel}+\mathrm{con}}}{S_{\left(s+c\right)\mathrm{top}}}$	$f_{\mathrm{steelbttom}}=-\frac{M_{\mathrm{steel}}}{S_{\mathrm{steelbottom}}}-\frac{M_{\mathrm{con}}}{S_{\left(s+c\right)\mathrm{bottom}}}+\frac{2M_{\mathrm{steel}+\mathrm{con}}}{S_{\left(s+c\right)\mathrm{bottom}}}$

**Table 2:** Comparison of the amount of steel required for ordinary and TO girder.

Cross-Section	Ordinary Girder (OG)			TO Girder (TO)			Area Ratio (TO/OG, %)
	Width (mm)	Height (mm)	Area (mm^2^)	Width (mm)	Height (mm)	Area (mm^2^)	
Top flange	800	40	32,000	300	15	4500	14.06
Web	26	3153	81,978	26	3146	81,796	99.8
Bottom flange	800	57	45,600	800	64	51,200	112.3
Total	-	-	159,578	-	-	137,496	86.2

**Table 3:** Dimensional parameters of the cross section (unit: mm).

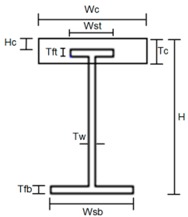	**Parameter**	***W*sb**	***W*st**	***T*fb**	***T*ft**
	Lower value	800	300	40	15
	Upper value	1000	400	60	25
	***T*w**	***H***	***W*c**	***T*c**	***H*c**
	26	3000	1500	300	150
	30	4000	2500	400	250
